# Modulation of H-reflex and V-wave responses during dynamic balance perturbations

**DOI:** 10.1007/s00221-023-06625-6

**Published:** 2023-05-04

**Authors:** Samuli Nevanperä, Nijia Hu, Simon Walker, Janne Avela, Jarmo M. Piirainen

**Affiliations:** 1 Sports Technology Program, Faculty of Sport and Health Sciences, University of Jyväskylä, Kidekuja 2, 88610 Vuokatti, Finland; 2 NeuroMuscular Research Center, Faculty of Sport and Health Sciences, University of Jyväskylä, Rautpohjankatu 8, PL35, 40700 Jyväskylä, Finland

**Keywords:** V-wave, H-reflex, Neural modulation, Dynamic condition, Dynamic balance

## Abstract

Motoneuron excitability is possible to measure using H-reflex and V-wave responses. However, it is not known how the motor control is organized, how the H-reflex and V-wave responses modulate and how repeatable these are during dynamic balance perturbations. To assess the repeatability, 16 participants (8 men, 8 women) went through two, identical measurement sessions with ~ 48 h intervals, where maximal isometric plantar flexion (IMVC) and dynamic balance perturbations in horizontal, anterior–posterior direction were performed. Soleus muscle (SOL) neural modulation during balance perturbations were measured at 40, 70, 100 and 130 ms after ankle movement by using both H-reflex and V-wave methods. V-wave, which depicts the magnitude of efferent motoneuronal output (Bergmann et al. in JAMA 8:e77705, 2013), was significantly enhanced as early as 70 ms after the ankle movement. Both the ratio of M-wave-normalized V-wave (0.022–0.076, p < 0.001) and H-reflex (0.386–0.523, p < 0.001) increased significantly at the latency of 70 ms compared to the latency of 40 ms and remained at these levels at latter latencies. In addition, M-wave normalized V-wave/H-reflex ratio increased from 0.056 to 0.179 (p < 0.001). The repeatability of V-wave demonstrated moderate-to-substantial repeatability (ICC = 0.774–0.912) whereas the H-reflex was more variable showing fair-to-substantial repeatability (ICC = 0.581–0.855). As a conclusion, V-wave was enhanced already at 70 ms after the perturbation, which may indicate that increased activation of motoneurons occurred due to changes in descending drive. Since this is a short time-period for voluntary activity, some other, potentially subcortical responses might be involved for V-wave increment rather than voluntary drive. Our results addressed the usability and repeatability of V-wave method during dynamic conditions, which can be utilized in future studies.

## Introduction

The H-reflex (Hoffmann-reflex) is an electrically induced, artificial reflex response that uses the same, naturally occurring monosynaptic reflex arc than muscle stretch reflex, even though not directly activating the muscle spindles (Aagaard et al. 2002; Schieppati 1987; Zehr 2002). H-reflex is used in studying the excitability of spinal pathways and synaptic actions on human spinal motoneurons (Pierrot-Deseilligny and Mazevet 2000). Since the beginning of the twentieth century, when Paul Hoffmann observed this artificial reflex response, the H-reflex has been widely used in various static and dynamic experimental settings. For example, Nielsen et al. (1993) and Mynark and Koceja (1997) examined the H-reflex responses with ballet dancers and control subjects in prone and standing conditions. The researchers found that the amplitude of H-reflex was highly dependent on both body posture and training background. The task-dependency of H-reflex was also well established by Hoffman and Koceja (1995) and Taube et al. (2008c), where both studies showed that H-reflex was suppressed when subjects’ vision was occluded during a balance task, seemingly influenced by altered presynaptic inhibition (PSI). Specifically, Hoffman and Koceja (1995) hypothesized that various supraspinal mechanisms, such as the descending drive from motor cortex, cerebellum and/or basal ganglia regulate the activity of the reflex arc via PSI. Thus, proprioceptive information may be modified and efficient neural control during demanding motor tasks could be obtained by lower gain from the reflex arc and greater supraspinally controlled movement.

A peripheral electrical stimulation of the nerve can be used to determine the magnitude of efferent motoneuronal output. Using supramaximal stimulation intensity (125–150% of maximal M-wave, i.e. Mmax) during ongoing voluntary muscle activity, a V-wave, which is an electrophysiological variant of the H-reflex, is observed (Aagaard et al. 2002; Upton et al. 1971). Here, the antidromic collisions of upstream travelling action potentials with downstream travelling efferent motoneuron action potentials allow the H-reflex to pass to the alpha-motoneurons and be detected in the EMG signal as a V-wave response. According to Grospretre and Martin (2014), the motor evoked potential responses (MEP) evoked by transcranial magnetic stimulation (TMS) during isokinetic muscle contraction of triceps surae muscles activate the same motor units as the V-wave, indicating that the V-wave is strongly influenced by voluntary drive. Also, according to studies by Del Balso and Cafarelli (2006) and El Bouse et al. (2012), submaximal-to-maximal isometric experiments showed that the amplitude of soleus and flexor carpi radialis V-waves were highly correlated with the amount of voluntary muscle activity during 50% to 100% and 20% to 100% of one-repetition maximum (1RM), respectively. However, motor control is organized differently during upright standing and during sudden perturbation with rapid muscle response compared to isometric and tonic muscle contractions. As several researchers (Jacobs and Horak 2007; Taube et al. 2008c; Valls- Solé et al. 2008) have proposed, subcortical structures in particular might play a crucial role in execution of rapid postural responses, even though the contribution of spinal, afferent (Schieppati and Nardone 1997) or transcortical, voluntary mechanisms (Shemmell 2015) cannot be ruled out.

So far the V-wave method, which depicts the magnitude of efferent motoneuronal output (Bergmann et al. 2013) has been used in experimental settings using both maximal (Aagaard et al. 2002; Duclay and Martin 2005; Vila-Cha et al. 2012) and submaximal (Del Balso and Cafarelli 2006; Pensini and Martin 2004) isometric voluntary muscle contractions and, despite some studies using the V-wave method in dynamic weight-bearing conditions (Alkjaer et al. 2013; Bergmann et al. 2013; Simonsen et al. 2012), studies using V-wave during balance perturbations or other dynamic balance tasks have not been conducted according to our knowledge. Furthermore, there are few dynamic balance studies published to-date, and most of them use either H-reflex (e.g. Piirainen et al. 2013) or TMS (e.g. Taube et al. 2006) to examine neural modulation. It is, thus, unclear how the V-wave behaves and reflects motor control during dynamic, weight-bearing conditions with various force levels and interaction with sensory and balance regulatory systems as well as how repeatable V-wave responses are, together with H-reflex, to study the modulation of human neural activity in dynamic conditions.

The main purpose of this study was to determine whether both H-reflex and V-wave methods are valid and feasible techniques also during dynamic balance task and to determine if neural modulation can be consistently identified by the amplitude of H-reflex and V-wave during an ongoing balance perturbation. Because of this purpose, the repeatability of the parameters characterizing the balance properties were also examined.

## Materials and methods

### Participants

Sixteen (8 men and 8 women; 30–45 years old, (height 175 ± 10 cm; weight 81 ± 16 kg; BMI 26 ± 3) healthy, daily-active participants with no prior severe musculoskeletal injuries were recruited into this study. Due to technical issues, balance data from four participants was lost, and the sample size for balance data was 12 (n = 12). At the beginning of the first measurement session, study details were described, and the participant signed a written consent document. Participants were also informed that participation was voluntary, and they had the right to withdraw from the measurements whenever they felt with no further consequences. This study was approved by the Ethics Committee of the local University and conducted according to the Declaration of Helsinki (2013), except for registration in a database.

### Design of the study

The two measurements days were implemented within 1–4 days and were scheduled at the same time of the day to avoid circadian changes in muscle activity (Mendonca et al. 2019). During each day, the protocol was identical with the exception that on the first visit, the participant’s weight (InBody 770 body composition analyzer, Cerritos, California, USA) and height were measured. The participants were requested to avoid heavy lower-body exercise 48 h and use of alcohol 24 h prior to measurements. Upon arrival to the lab, participants carried out the first balance test familiarization, which included 16 uniformly accelerating anterior–posterior perturbations induced by the balance platform with horizontal translation by a linear motion. The velocity, acceleration and the amplitude of the balance platform perturbations were identical with the perturbations of the actual measurements. After placing the EMG electrodes, the participant performed an 8-min warm-up on a Monark LC7TT-cycle ergometer (Monark, Vansbo, Sweden). The resistance was set so that the participant was able to maintain a cadence of approximately 90 rpm and talk easily during the warm-up, giving an approximate cycling power of 80 watts. After warm-up, measurements were completed in the following order: 1) M-wave recruitment curve during quiet standing, 2) maximal plantar flexion isometric voluntary contraction (IMVC), 3) V-wave measurements during IMVC, 4) the second balance test familiarization, 5) H-reflex and V-wave measurements during balance perturbations and 6) dynamic balance perturbation test. Total duration of one measurement set was approximately 2 h. All measurements have been described more detailed in the following sections.

### EMG preparation and data collection

After careful preparation of the skin (shaving, abrasion with fine sandpaper and cleaning with alcohol) following SENIAM instructions (Hermens et al. 1999), round, self-adhesive EMG-electrodes (Blue Sensor, Ag/AgCl, Ø28 mm, Ambu A/S, Ballerup, Denmark) were placed bipolarly (with inter-electrode distance of 2 cm) on the right leg of the participant’s soleus muscle (SOL) in line with the Achilles tendon two centimeters below the gastrocnemius muscle. To improve the repeatability between days, the exact placements of EMG-electrodes were marked with a permanent marker. Finally, the required impedance of < 5 kΩ was checked and the electrodes were taped to the skin with sports tape (Leukoplast, Hamburg, Germany) to avoid possible movement of the electrodes. The EMG-signal (muscle activity) was collected using a Neurolog system (NeuroLog NL900D; Hertfordshire, United Kingdom), amplified 1000x, band-bass filtered to 10–500 Hz and the sampling rate was set 1 kHz with CED analog-to-digital converter (CED Power 1401; CED Ltd., Cambridge, England). The data was recorded with Spike 2 5.14-software (CED Ltd., Cambridge, England).

### M-wave recruitment curve

To implement the recruitment curve and elicit the maximal M-wave (M_max_) from SOL, an anode (5 × 8 cm) was placed over the patella and a cathode (1.5 × 1.5 cm) was placed in the popliteal fossa to stimulate the right leg tibial nerve using a Digitimer stimulator (Constant current stimulator DS7A, Digimeter Ltd, Welwyn Garden City, England) with a 200 μs square pulse. The participant was instructed to stand still, and the best possible stimulation spot was located by moving a temporary cathode over the popliteal fossa until the highest M-wave peak-to-peak amplitude was observed with constant stimulation intensity. In addition, the constant shape of the H-reflex was carefully monitored in different stimulation intensities. After the stimulation spot determination, the stimulating cathode was placed and firmly tightened under pressure with an elastic bandage rolled over the knee joint to avoid electrode movement. To determine M_max_, stimulations were delivered with 10 s intervals to avoid post-activation depression (Crone and Nielsen 1989) and the stimulation intensity was increased by 1–5 mA until M_max_ was reached. To ensure a maximal M-wave, at least one supramaximal stimulation (150% M_max_) was given. The peak-to-peak amplitude of M-wave was manually determined using Spike 2 5.14-software.

### Plantar flexion IMVC and V-wave

IMVC was conducted using a custom-build dynamometer (University of Jyväskylä, Jyväskylä, Finland) where the participant sat in a position with a hip, knee and ankle angle of 110°, 180° and 90°, respectively (180° = fully extended). The participant sat on the bench with his/her back firmly against the back rest, harness was tightened, the bench was adjusted, and the distance of the bench (cm) was marked to ensure the same settings between days. Before the actual performances, the participant was instructed to hold on the grips with his/her hands, to set his/her right foot against the platform to the same place with each performed contraction and to take a deep breath just prior to go-signal. At this point, 4–5 fast, submaximal warm-up contractions were performed. To determine IMVC and maximal rate of force development (RFD) the participant performed three maximal, 3-s all-out plantar flexions with 1.5-min intervals. If the third performance was at least 5% higher than the second best, 1–3 additional trials were performed to ensure maximal force. The data was collected using CED Power 1401A/D converter and Spike 2 5.14-software with sampling rate of 1 kHz.

After IMVC, the V-wave was measured from the right leg SOL using the same IMVC setup. The lowest acceptable force level during these tests was set to 90% of the IMVC (Aagaard et al. 2002) and five successful V-waves were measured with a supramaximal (150% M_max_) electrical stimulation applied to the tibial nerve. Stimulation was manually applied immediately after the force reached the 90% level to standardize the phase of the stimulation. The performance was considered successful if: 1) the participant could reach and exceed the level of 90% of IMVC, 2) the stimulation was timed and elicited in an ascending force curve after crossing the 90% force limit, and 3) the V-wave was visually noticeable from the EMG signal. Altogether, the participants performed 5–9 contractions to achieve 5 successful performances.

### Dynamic balance perturbations

Perturbation parameters were determined and executed (acceleration 2.5 m/s^2^, maximum velocity 30 cm/s and displacement 30 cm in anterior–posterior direction) with LabVIEW (National Instruments, Austin, USA) and IndraWorks software (Bosch Rexroth, Lohr am Main, Germany). The platform moved in a randomized order, not returning to its original position until after the last trial.

**Fig. 1:**
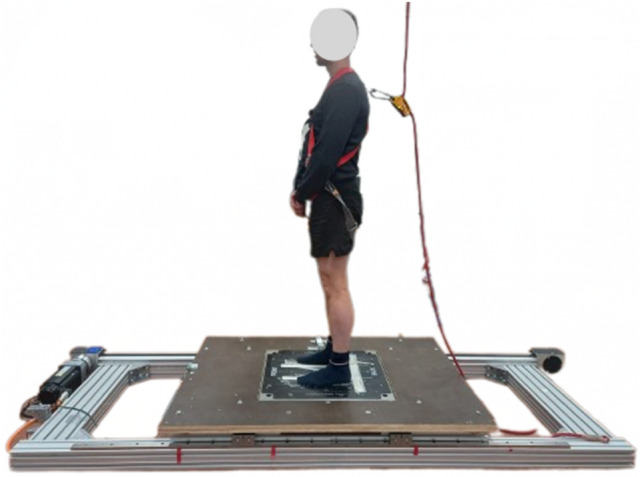
An illustration of the custom-build balance platform, which induced anterior–posterior perturbations with horizontal translation by a linear motion. The participant stood on the black, square-shaped force plate mounted on the center of the platform. Participants wore a safety harness, which was used to prevent possible falling

The balance platform (BT4 balance platform; HUR Labs, Tampere, Finland) was mounted to a modified custom-built (Piirainen et al. 2013; Walker et al. 2020) dynamic balance platform (University of Jyväskylä, Jyväskylä, Finland) shown in Fig. 1. The total length of the device was 3 m and force plate dimensions were 106.5 cm × 42.0 cm × 2.7 cm. The platform was controlled from the main center (Bosch Rexroth, IndraDrive Cs, Germany, Lohr am Main) using a servomotor (Bosch Rexroth, 3-phase synchronous pm-motor, Germany, Lohr am Main), which produced displacements via a motor-driven belt. The balance data was collected using Coachtech-software (University of Jyväskylä, Jyväskylä, Finland) with a sampling rate of 400 Hz. Raw force signals were low-pass filtered using Finite Impulse Response (FIR) filters with 25 Hz as cut-off frequency. Center-of-pressure (COP) in anterior–posterior direction was calculated using the following formula: COPy = ((Flf + Frf) × 0.26—(Flr + Frr) × 0.26) / (Flf + Frf + Frr + Flr), where lf = left front, rf = right front, rr = right rear, lr = left rear and 0.26 m is the sensor distance from the middle line. COP was filtered using the same low pass FIR filter with 7 Hz. The platform movement was triggered when COP was inside ± 5 mm level from the zero level for at least 1 s. This approach ensured that the participant was always standing straight and still without any anticipation for the upcoming perturbation.

During the dynamic balance tests, the participants used a safety harness (CAMP Empire, Perth, Australia) to ensure that they did not fall. The participant was instructed to stand still and keep their legs straight (knees locked) and on predefined spots, hands in front of him/her and to fixate their eyes on the dot on the wall (distance 3.0 m) to stabilize the sense of sight. During the balance sets, the participant was asked to stand as relaxed as possible and to react to the sudden perturbations with the most efficient manner and to restore equilibrium as quickly and efficiently as possible. Before actual measurements, participants performed two familiarization sets of 16 perturbations identical to actual measurement sets: one set before electrode set-up and one set immediately before the actual measurements. This was done to reduce the anxiety and to get familiarized with the balance platform and consequently reduce the possible excessive stress-induced muscle tension and muscle activity.

During the dynamic balance perturbations, the rectangular clock pulse, which controlled the movement of the platform, was used as a trigger for electrical stimulations and the constant delay between the platform clock pulse and ankle movement was 25 ms (Hu et al. 2022). Both SOL H-reflex and V-wave responses were generated at 4 different latencies: 40, 70, 100 and 130 ms from the beginning of the ankle movement. The latencies were chosen according to literature and to coincide with short latency (SLR; 30–50 ms) (Corden et al. 2000), medium latency (MLR; 65–100 ms), long latency (LLR; ≥ 100 ms) (Miranda et al. 2019) and voluntary responses (Taube et al. 2008c). For each latency, a set of 16 perturbations (8 anterior and 8 posterior perturbations) was performed in random order with 6–12 s intervals and a 2-min sitting break was conducted after every third set. The stimulations were delivered during each perturbation, regardless of the direction of perturbation, but only posterior perturbations, when responses in SOL are induced, were analyzed in this study.

During the SOL H-reflex measurements, the stimulation intensity was manually adjusted to a target level of 5% ± 2.5% of the maximal M-wave measured during standing rest. During the V-wave recordings, the stimulation intensity was set to supramaximal (150% M_max_) with no manual adjustment of stimulation intensity between trials. One set of perturbations was performed and recorded without stimulation. For practical reasons, all sets of H-reflex were measured first, before the V-wave sets, and the non-stimulated set was always last. However, the delays for H-reflex and V-wave measurements were randomized between participants and the order of execution of sets were matched between days for each individual participant. During the H-reflex measurements, a minimum of 5 successful trials for each set of 8 posterior trials were recorded, and if the number of accepted trials were lower than 5, an additional perturbation set was measured. Participants were exposed to a total of 176–212 perturbations during one session, including familiarization sets.

### Data analyses

IMVC (N) was used to determine the individual’s maximum force capacity and therefore the highest point of force curve (peak-to-peak) was analyzed. RFD (N/s) was analyzed by using an epoch of 200 ms following the onset of force production and calculated by dividing the peak-to-peak amplitude of force (N) within 200 ms time window with the time period (0.2 s). The peak-to-peak amplitudes of maximal M-waves (M_max_) and V-waves during isometric contractions (V_iso_) were analyzed and consequently the V_iso_/M_max_ -ratio was calculated (average of 5 successful performances). To verify identical pre-stimulus (prestim) conditions between day 1 and day 2, the average background EMG 50 ms prior to V-wave stimulations (EMG_IMVC_PRESTIM_) were calculated from all 5 stimulated IMVC performances, which were thereafter normalized to M_max_ standing rest. Consequently, the values were averaged within all participants.

From each balance perturbation, SOL background EMG, peak-to-peak M-waves, H-reflexes and V-waves were analyzed. The successful H-reflexes during balance perturbations (H_bal_) were normalized to M_max_ standing rest. Consequently, the H_bal_(%M_max_) was calculated. The V-wave during balance perturbations (V_bal_) was normalized to adjacent M_max_ and consequently the V_bal_(%M_max_) was calculated. H_bal_ was used to quantify spinal excitability, whereas V_bal_ was used to quantify the amount of efferent motoneuronal output. Thus, to depict the ratio of spinal and efferent motoneuronal output at different delays, the percentage of V_bal_/H_bal_ was calculated and reported as V_bal_(%H_bal_) (see also TMS/H-reflex ratio: Duclay et al. 2014).

SOL background EMG RMS (Root Mean Square; RMS) was calculated 30 ms prior to stimulations over each delay and both recordings (H-reflex and V-wave) and reported as EMG_bal_prestim_. Two other parameters of SOL background EMG were analyzed from the last set where no simulations were given: (1) the raw EMG for each participant was calculated as an average of 8 posterior perturbations over a 25–190 ms time window (i.e. from the beginning of ankle movement), then rectified, smoothed (0.002) and normalized to each participant’s individual M_max_. The normalized EMG curves were averaged between all participants and reported as EMG_bal_(%M_max_). (2) The RMS of EMG, which was calculated over 20 ms time windows, ± 10 ms of the specific stimulation time-point and reported as EMG_bal_raw20_. All SOL background EMG measurements were normalized to individual M_max_ at standing rest.

From the balance data, we analyzed the average height and weight normalized COP peak-to-peak displacement (mm/(height([m])*weight[kg])) and swaying velocities ((mm/s)/(height[m])*weight[kg])) (Chiari 2002) from preparatory phase (PRE), active phase (ACT) and recovery phase (REC). PRE-phase was analyzed over a 1 s time window prior to platform movement, ACT-phase over a 1.2 s (total duration of the platform movement) time window following the onset of the platform movement and the REC phase was analyzed over a time window of 1 s from the end of the plate movement and at this phase the participant was still leaning forward and gaining back the equilibrium.

### Statistical analyses

The result visualizations were performed with Prism GraphPad-software version 9.1.0 (GraphPad software, San Diego, USA). For statistical analyses, SPSS 26.0-software was used. First, the means and standard deviations (SD) for every individual participant were calculated. The normality of data was analyzed with the Shapiro–Wilk-test before further analysis. Intraclass Correlation Coefficient (ICC) was used to determine the repeatability between days and ICC was interpreted as follows: ICC 0.11 to 0.40 = slight, ICC 0.41 to 0.60 = fair, ICC 0.61 to 0.80 = moderate and ICC 0.81 to 1.0 = substantial (Mendonca et al. 2019; Solstad et al. 2011). To analyze H-reflex and V-wave parameters, two-way repeated measures ANOVA was used, where main effects of delay (4) and day (2) were analyzed. In addition, delay × day interaction was tested. If sphericity was not assumed, the Greenhouse–Geisser formula was used to adjust degrees of freedom. If a significant main effect was found, LSD pairwise comparisons were performed. Effect sizes were also tested using partial eta squared (η_p_^2^), where 0.02, 0.13 and 0.26 were considered as small, medium and large effect, respectively. Between-day differences in IMVC, RFD, COP amplitude of swaying velocity and maximum peak-to peak amplitude of COP were tested using paired samples t-tests. Results were significant if the p-value was less than 0.05.

## Results

### Modulation of H-reflex and V-wave responses

**Fig. 2:**
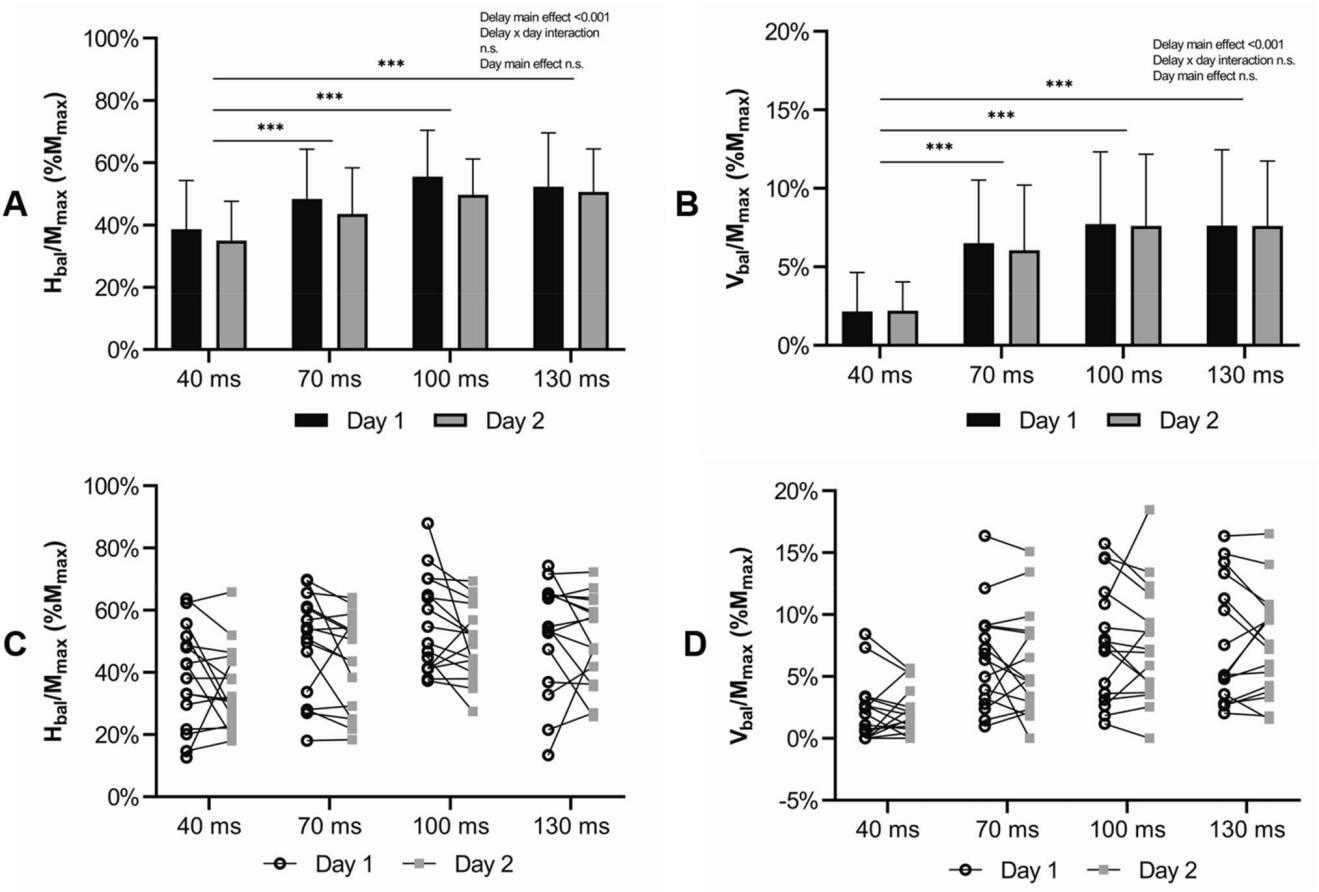
**A** Averaged H_bal_ and **B** averaged V_bal_ with standard deviations in all four delays and from both measurement days of balance perturbations. The asterisks (***p < 0.001) indicate a significant difference between delays. Individual changes in (**C**) H_bal_ and (**D**) V_bal_ in all delays from day 1 and 2. The line connects the day 1 and day 2 results of each individual

**Fig. 3:**
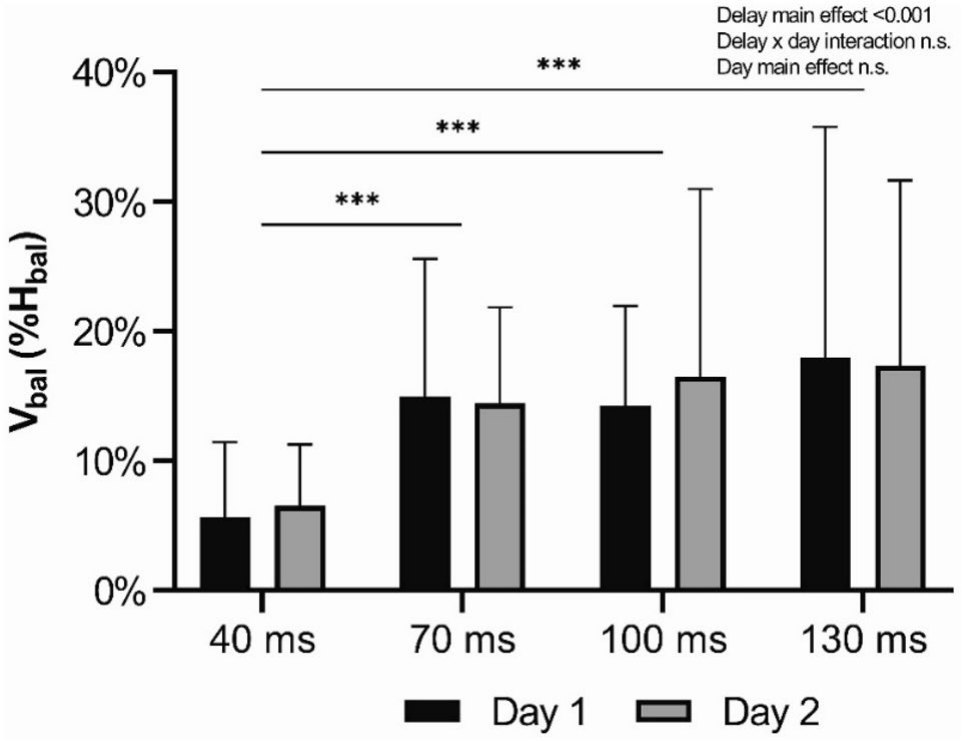
(V_bal_/M_max_) / (H_bal_/M_max_) -ratio depicted with standard deviation from each delay (40, 70, 100 and 130 ms) and from both measurement days of balance perturbations. The asterisks (***p < 0.001) indicate a significant difference between delays. No delay × day interaction or day main effect was observed

H-reflex responses are shown in Fig. 2A. A main effect of delay was observed (F = 36.230, p < 0.001, η_p_^2^ = 0.547) with no delay × day interaction (F = 0.539, p = 0.657, η_p_^2^ = 0.018) or main effect of day (F = 0.659, p = 0.423, η_p_^2^ = 0.021). H-reflex was significantly lower (p < 0.001) during the 40 ms delays compared to latter delays of 70, 100 and 130 ms. As shown in Fig. 2B, V-wave behaved very similarly, as the averaged V_bal_ increased towards latter delays. A main effect of delay was observed (F = 18.259, p < 0.001, η_p_^2^ = 0.378) with no delay × day interaction (F = 0.03, p = 0.993, η_p_^2^ = 0.001) or main effect of day (F = 0.017, p = 0.896, η_p_^2^ = 0.001). Following pairwise comparisons, V-wave was significantly lower (p < 0.001) during the delay of 40 ms compared to the latter delays of 70, 100 and 130 ms. Neither H-reflex nor V-wave showed significant differences between the delays of 70, 100 and 130 ms. V_bal_(%H_bal_) shown in Fig. 3 was significantly increased at the delay of 70 ms compared to the earlier delay of 40 ms. A main effect of delay was observed (F = 7.679, p < 0.001, η_p_^2^ = 0.204) with no delay × day interaction (F = 0.142, p = 0.868, η_p_^2^ = 0.005) or main effect of day (F = 0.033, p = 0.856, η_p_^2^ = 0.001).

### Between-day repeatability of H-reflex and V-wave

The two separate, individual measurement days showed substantial repeatability in V-wave delays of 70, 100 and 130 ms, whereas the 40 ms delay showed moderate ICC. The repeatability of H-reflex showed moderate ICC in delay of 40 ms, substantial ICC in latency of 70 ms, fair ICC in delay of 100 ms and moderate ICC in delay of 130 ms (Table 1). On an individual level, the between day changes in H-reflex and V-wave are presented in Fig. 2 C and D, respectively.

**Table 1:** Between-day repeatability of H-reflex (H) and V-wave (V) with delays of 40 ms, 70 ms, 100 ms and 130 ms after ankle movement

Delay	ICC	95% CI	p-value	Correlation
H40	0.632	− 0.052–0.872	0.031*	Moderate
H70	0.855	0.585–0.949	<0.001***	Substantial
H100	0.581	− 0.199–0.854	n.s.	Fair
H130	0.728	0.222–0.905	0.008**	Moderate
V40	0.774	0.354–0.921	0.003**	Moderate
V70	0.869	0.624–0.954	<0.001***	Substantial
V100	0.885	0.671–0.960	<0.001***	Substantial
V130	0.912	0.749–0.969	<0.001***	Substantial

*ICC* lntraclass correlation coefficient, *CI* Confidence Interval, *n.s.* not significant

### SOL muscle activity

**Fig. 4:**
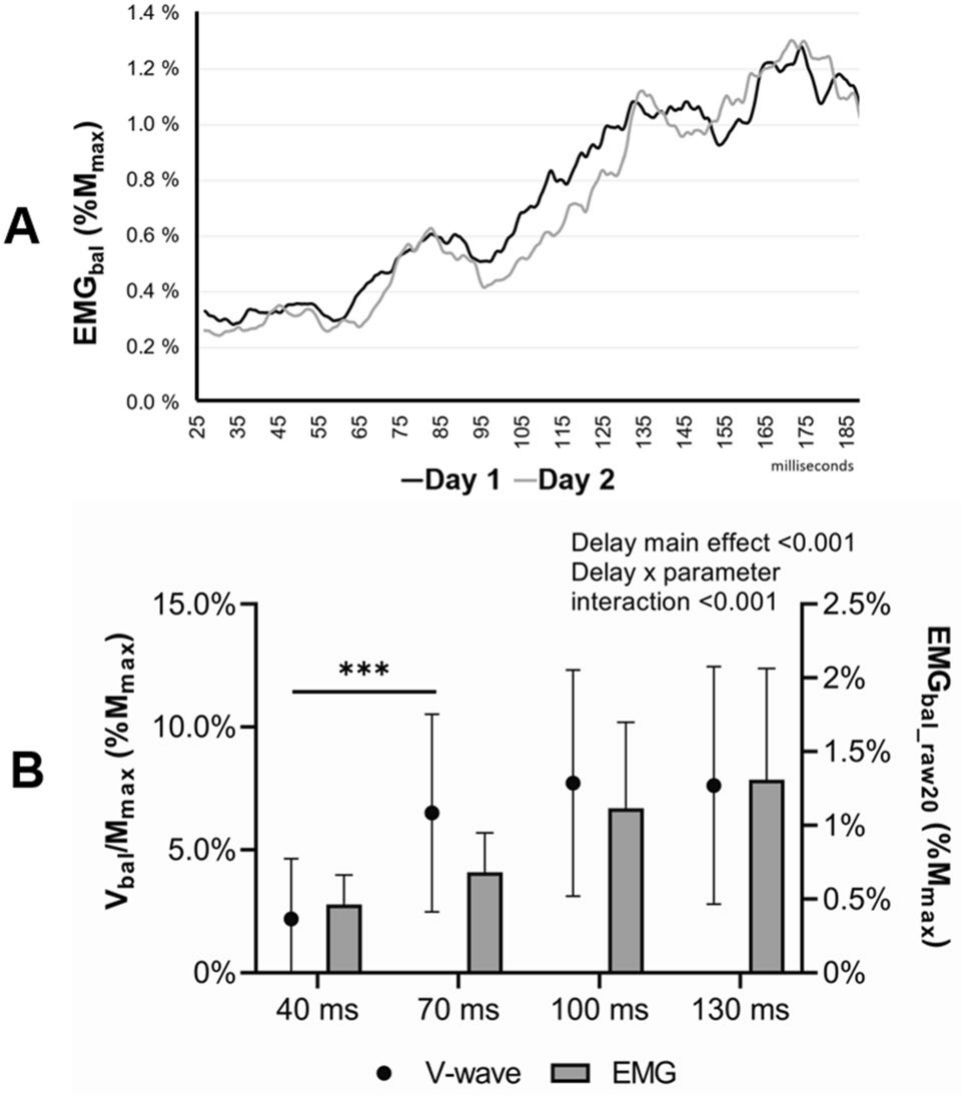
**A** The averaged, rectified and smoothed SOL muscle activity expressed as EMG_bal_ from both days from the beginning of ankle movement until 190 ms. Black line represents day 1 and grey line represents day 2. Substantial repeatability (ICC = 0.982) was found between days. **B** Both V-wave (left y-axis; black dots) and muscle activity (right y-axis; grey bars) presented at different delays from day 1. EMG_bal_raw20_ was calculated in 20 ms time windows: ± 10 ms of the onset of each stimulation delay. A significant delay × parameter (EMG and V-wave) interaction (p < 0.001) and main effect (p < 0.001) was observed. Although V-wave increased significantly between delays of 40 ms and 70 ms (p < 0.001), this was not observed in SOL muscle EMG (p = 0.685)

Muscle activity normalized to maximal M-wave of SOL is presented in Fig. 4A over a 25–190 ms time window. This averaged EMG_bal_(%M_max_) of participants (n = 15) showed substantial between-day repeatability (ICC = 0.982). In averaged EMG_bal_raw20_ a main effect of delay was observed (F = 63.770, p < 0.001, η_p_^2^ = 0.680) with no delay × day interaction (F = 0.274 p = 0.763, η_p_^2^ = 0.009) or main effect of day (F = 0.384, p = 0.540, η_p_^2^ = 0.013). Statistically significant (p < 0.05 – p < 0.001) increase was observed between all delays. The Fig. 4B depicts the muscle activity (EMG_bal_raw20_) alongside with V-wave activity from day 1. Between the EMG_bal_raw20_ and V-wave responses, a significant delay × parameter interaction (F = 7.732, p < 0.001, η_p_^2^ = 0.205) and main effect of delay (F = 12.848, p < 0.001, η_p_^2^ = 0.300) was observed. Whereas V-wave showed statistically significant (p < 0.001) increase between delays of 40 ms and 70 ms, in EMG_bal_raw20_ this was not observed (p = 0.685). In EMG_bal_prestim_ during H-reflex recordings, a main effect of delay was observed (F = 58.045, p < 0.001, η_p_^2^ = 0.659), with no delay × day interaction (F = 0.155, p = 0.926, η_p_^2^ = 0.005) or main effect of day (F = 1.217, p = 0.279, η_p_^2^ = 0.039). During V-wave recordings, the values were (F = 86.042, p < 0.001, η_p_^2^ = 0.741), (F = 0.260, p = 0.854, η_p_^2^ = 0.009) and (F = 0.403, p = 0.531, η_p_^2^ = 0.013), respectively. There was statistically significant difference between delays (from p = 0.004 to p < 0.001) in both H-reflex and V-wave recordings, but not between delays of 40 ms and 70 ms (p = 0.939 and p = 0.825), respectively.

### IMVC, RFD and Dynamic Balance control

**Fig. 5:**
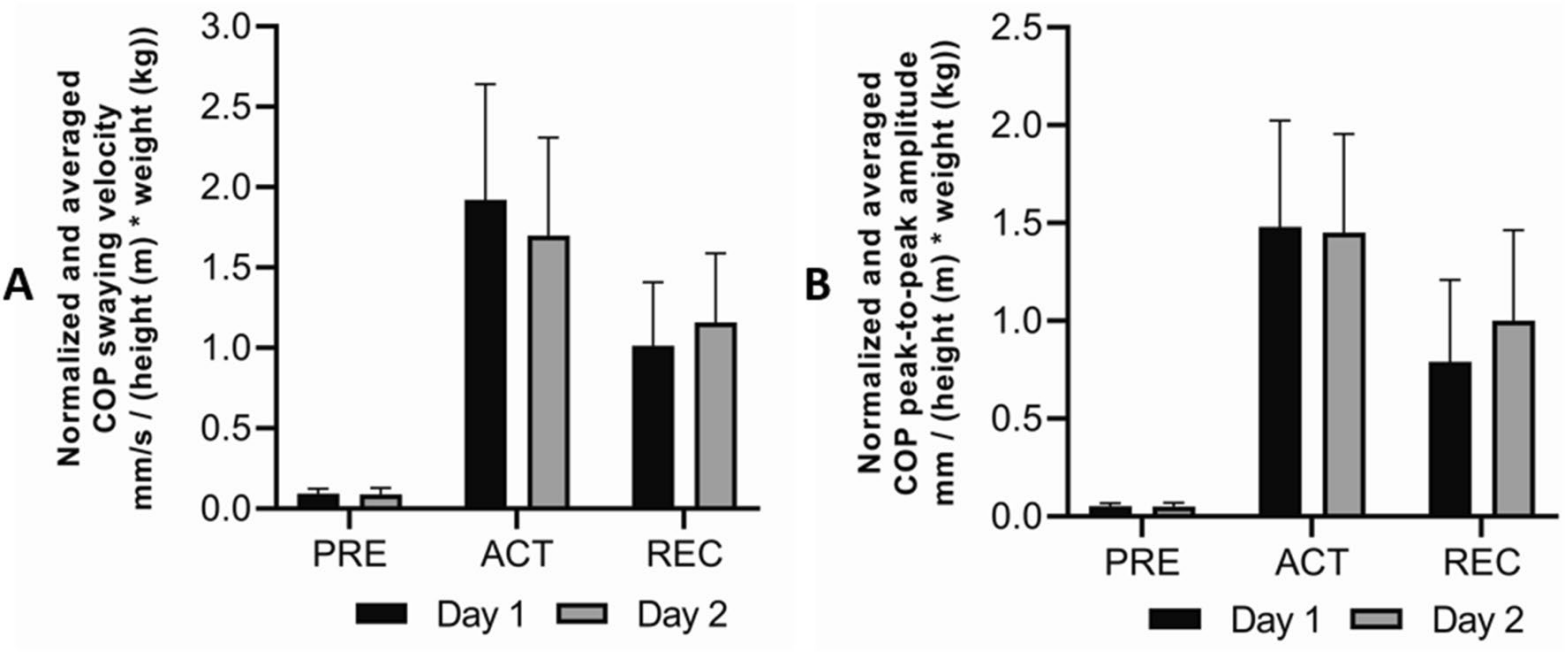
**A** Normalized COP swaying velocity and **B** normalized COP amplitude (peak-to-peak max) averaged with participants (n = 12) and expressed from day 1 (black) and day 2 (grey). No differences were observed in normalized COP swaying velocities or in COP amplitudes

No between-day differences were observed in plantar flexor IMVC (Day 1; 1941 ± 371 N, Day 2; 2000 ± 352 N), RFD (Day 1; 4980 ± 1692 N/s, Day 2; 4908 ± 1349 N/s). Similarly, the EMG_IMVC_PRESTIM_ indicated equivalent EMG levels between measurements. The EMG_IMVC_PRESTIM_/M_max_ -ratio was 0.036 ± 0.01 and 0.033 ± 0.008 (p = 0.406) from day 1 and day 2, respectively. Also, no differences were observed in normalized COP swaying velocities or COP amplitudes (Fig. 5A and B, respectively) between day 1 and day 2. Furthermore, M_max_ values (between 5.81 ± 1.34 and 6.06 ± 1.22 mV) were constant between days in standing rest (p = 0.843) and in all balance perturbation conditions (between p = 0.752 and p = 0.982).

## Discussion

The main purpose of this study was to investigate neural modulation during dynamic balance perturbations using H-reflex and V-wave responses, and secondarily to assess the between-day repeatability of the H-reflex and V-wave. The H-reflex and V-wave responses behaved consistently and systematically during perturbations, and both made a significant increase in amplitude from 40 to 70 ms after ankle movement. The V-wave showed excellent between-day repeatability during dynamic balance tests, whereas the H-reflex showed more inconsistent repeatability.

### Modulation of H-reflex and V-wave responses during perturbations

H-reflex amplitude was significantly higher at a delay of 70 ms compared to delay of 40 ms and maintained a similar amplitude at latter delays. It has been shown, that during dynamic, posterior perturbations H-reflex was stable between delays of 10 and 90 ms (Piirainen et al. 2013). On the other hand, H-reflex has been shown to increase at around 86 and 100 ms after ankle movement during posterior perturbation (Taube et al. 2006; Miranda et al. 2019, respectively). The increase of H-reflex at longer delays could be a result of summation of both proprioceptive activity and descending drive, which both increase spinal excitability.

As shown in Fig. 2A and B, both H-reflex and V-wave indeed made a substantial increase in amplitude at 70 ms delay. However, the V_bal_(%H_bal_) (Fig. 3) clearly showed that the contribution of V_bal_ increased towards latter delays. Thus, this indicates that the efferent motoneuronal output was significantly increased between delays of 40 ms and 70 ms. One possible explanation for the increased motoneuronal output could be increased descending drive. Although, in general, a shift from spinal-regulated motor control to more supraspinal-regulated control at longer delays during sudden perturbations is somewhat logical. What is more interesting, is that the modulation in our study showed substantially higher contribution of V-wave already at 70 ms after ankle movement. As Taube et al. (2006) have demonstrated, the earliest transcortical responses measured with TMS were detected already at 86 ms delay after ankle movement and the voluntary activity and the activity of primary motor cortex were enhanced at around > 120 ms delay (Taube et al. 2008c). Also, one dynamic balance perturbation study, which was implemented with TMS, showed that the MEP responses were significantly augmented as late as 140 ms after ankle movement (Hu et al. 2022).

The significant increment in V-wave amplitude already at 70 ms after ankle movement raises the question whether the subcortical segments, such as the brainstem, cerebellum or basal ganglia contributed to increased V-wave. As Jacobs and Horak (2007) and Shemmell et al. (2010) have proposed in their reviews, these brain segments are responsible for the prior postural responses during sudden external perturbations. The function of subcortical structures, such as the brainstem and reticular formation, which have inputs to lower motoneurons via reticulospinal tract, can be studied indirectly and noninvasively with the StartReact-procedure (Atkinson et al. 2022; Baker and Perez 2017; Honeycutt et al. 2013). In the study by Valls-Solé et al. (1998), subjects were instructed to react to a visual “go”-signal as fast as possible. In some trials, the subjects were exposed to additional external stimuli, a loud noise called startle stimulus, which leads to a primitive, involuntary response. The study revealed that the startle stimulus could halve the latency of voluntary response and in some cases the reaction times were even shorter than the theoretical, calculated minimum time for cerebral cortex to process sensory information. The authors stated that the movement pattern might be stored in the brainstem and could be triggered without the command of cerebral cortex through the reticular formation and via reticulospinal tract to lower motoneurons. This phenomenon of facilitation of subcortical structures as a result of a startling auditory stimulus was also stated in the review of Atkinson et al. (2022). In addition, Valls-Solé et al. (2008) argued in their review that subcortical mechanisms play a crucial role in preparation and execution of fast responses, whereas the higher centers are under inhibitory control until the movement is fully prepared. To conclude, such neural, subcortically mediated mechanisms might also be visible as higher V-wave amplitudes during rapid postural responses to balance perturbations at very early latencies, as was demonstrated in our results. However, the origin of an increase in V-wave amplitude is under debate and has some uncertainty (McNeil et al. 2013), and unfortunately no studies which would support our view of the influence of reticular formation and reticulospinal tract in V-wave recordings have not been done.

Nevertheless, the significant increase in V-wave activity in our study coincides with the controversial MLR, which has been proposed to be generated at spinal level (Taube et al. 2008a), assumably through slow group II fibers (Grey et al. 2001; Schieppati and Nardone 1997; Uysal et al. 2009), but it has also been suggested, that MLR is partly mediated by the transcortical loop in upper limbs (Matthews et al. 1990), but not in lower limbs, as there was extra-facilitation of MEP only during LLR, but not during SLR or MLR (Petersen et al. 1998). However, the organization of neural control appears far more complex. EMG_bal_raw20_ showed statistically significant delay × parameter interaction, and whereas V-wave increased significantly between delays of 40 ms and 70 ms, this was not the case with EMG_bal_raw20_, meaning that there was clear V-wave activity independent from EMG activity.

Consequently, the timing and role of subcortical structures in motor control remains unclear. As Shemmell (2015) has proposed, involuntary and voluntary responses overlap substantially and the responses to several tasks and posture-regulation are generated from multiple inputs, such as cerebellum, reticular formation and primary motor cortex. This cooperation of dexterity-based transcortical reflex loop together with the less flexible, but faster subcortical pathways allow the human species to react rapidly, but also with adequate precision.

### Repeatability of H-reflex and V-wave responses during perturbations

The results showed that especially the V-wave delays of 70, 100 and 130 ms showed substantial repeatability between days (ICC≥0.869), whereas the repeatability of V-wave at delay of 40 ms showed moderate repeatability (ICC = 0.774). Thus, the repeatability of V-wave was excellent overall during a dynamic balance situation and the results are equivalent to test–retest studies executed during static, isometric settings (Mendonca et al. 2019; Solstad et al. 2011).

The repeatability of H-reflex in our study, however, was weaker and more inconsistent (ICC = 0.581–0.855) compared to V-wave. The repeatability of H-reflex has been examined in various previous studies. For example, the study by Hayes et al. (2009) showed substantial test–retest repeatability of SOL H-reflex in supine position and Al Amer et al. (2020) demonstrated moderate-to-substantial repeatability in three different sitting postures measured within one day. Also, the study by Mynark (2005) showed substantial SOL H-reflex repeatability among young subjects in both supine and standing position, whereas older adults showed weaker repeatability during standing, probably due to greater body sway. Furthermore, the study by Hopkins et al. (2000) revealed that H-reflex intersession repeatability from SOL was better in supine (ICC = 0.938) than in standing (ICC = 0.803) position. This finding might address the fact, that the instability during weight-bearing conditions can lead to greater variance in H-reflex responses causing a challenge to standardize measurement conditions. It may be proposed that the weaker repeatability of H-reflex in our study was a consequence of low number of trials, as only 5–8 perturbations were averaged for an individual subject in each delay conditions. Al Amer et al. (2020), Handcock et al. (2001) and Hopkins et al. (2000) all stated that 4–5 responses of H-reflex was sufficient to obtain moderate-to-substantial SOL H-reflex repeatability. However, it must be recognized that none of these studies were implemented in dynamic conditions and might underline the task-dependency and explain the conflict between the studies.

### Repeatability of muscle activity (EMG) and balance regulation

The average muscle activity (EMG_bal_) during balance perturbations showed substantial repeatability between separate measurement days. The lack of tibialis anterior muscle (antagonist) activity raised a question whether there were any changes in movement patterns and/or agonist–antagonist muscle coactivation between days. This, however, probably was not the case as SOL muscle activity was very consistent between days, as shown in Fig. 4A. The SOL muscle EMG pattern was similar to that in the study of Wälchli et al. (2017), which showed minor increment in muscle activity at MLR, and significantly increasing at LLR. Also, in EMG_bal_raw20_ no statistically significant changes were observed between days or in delay × day interaction. When observing the balance data, statistical differences neither in normalized COP amplitude nor in normalized COP swaying velocity were observed between days. These parallel and uniform between-day muscle activity and balance regulation support the fact that the conditions were constant for measuring the repeatability of SOL H-reflex and V-wave responses and it can be assumed that no substantial changes in motor learning occurred between the measurement days.

### Usability and future directions

In contrary to TMS, which measures mostly the excitation of corticospinal tract, the V-wave might be able to gather more diverse efferent motoneuronal output, including descending drive and maybe detect the automated, subcortical responses during sudden, rapid perturbations that even bypass the cerebral cortex. However, it is not possible to specify the exact locus of these neural events solely with the V-wave method, and for this reason, we can only make assumptions whether the brainstem and other subcortical areas of the brain contribute to increase in V-wave responses and were indeed responsible for motor control in the early stage of the perturbations, or whether the slow group II afferents contributed to V-wave increment. However, the fact that our study showed substantial increase in efferent motoneuronal output (which might reflect the magnitude of descending drive) already at 70 ms delay after ankle movement, leaves a question of in what order the cortical and subcortical mechanisms regulate the motor control during sudden balance perturbation. In the future, by using the StartReact-procedure or by combining for instance EEG- or different TMS-measurements (MEP, CMEP) together with V-wave method, it could be possible to broaden the understanding of V-wave mechanisms and to estimate the magnitude and contribution of separate brain areas to dynamic balance perturbations. This, in turn, might give some confirmation to the contribution of subcortical areas in motor control.

Most of the current balance studies have been implemented during quiet standing, but static balance tests do not adequately stress the postural control system and does not develop a proper understanding of the motor control theory (Koceja et al. 1995). Furthermore, to challenge the existing methods, H-reflex measures only spinal excitability, which, however, is influenced by several, both central and peripheral mechanisms via presynaptic inhibition, whereas TMS has technical challenges in dynamic protocols, such as the long preparations when finding the hot-spot and stabilizing the coil e.g. with modified helmets. Therefore, V-wave may be an optional method for measuring motor control and total magnitude of motoneuronal output (also affected by descending drive) during dynamic balance conditions, and for example studying the acute effects of fatiguing tasks where measurement must be done as fast as possible after the loading to avoid effects of recovery. 

### Limitations and methodological considerations

The weaker repeatability of H-reflex was presumably a consequence of too few trials and at least 10 successful H-reflex perturbations might have improved the repeatability. Although several studies have showed that as few as 4–5 responses are enough to obtain a good repeatability during prone or quiet standing, dynamic conditions stress the postural system more vigorously and this might influence the stability of both M-wave and H-reflex. However, increasing the number of perturbations and elongating the measurement session can lead to fatigue. To avoid this and to keep the duration of one session at approximately 2 h, we decided upon four delay conditions for both H-reflex and V-wave.

Furthermore, we chose the delays of stimulation according to previous results and our own pilot tests. We wanted the stimulations to coincide with SLR, MLR and LLR and to induce voluntary responses. Regardless of our planning, as shown in the EMG_bal_ data in Fig. 4A, we missed the highest point of muscle activity, which on average occurred at approximately 175 ms after ankle movement. Nevertheless, as Taube (2008c) has stated, cortical activity should be observed at approximately 120 ms after ankle movement and according to this, our latest delay (130 ms) should have had enough time to activate primary motor cortex and to elicit voluntary responses. Yet, having 5 delays for both H-reflex and V-wave could be worth to consider in the future studies to detect the voluntary responses at very long delay, even though the risk of fatigue remains.

## Conclusions

This study examined neural modulation measured with H-reflex and V-wave responses during a dynamic balance test. Both the amplitude of H-reflex and V-wave enhanced significantly 70 ms after ankle movement. The ratio of V_bal_(%H_bal_) showed a clear increase towards latter delays, which may indicate that increased activation of motoneurons occurred due to changes in descending drive.

The study also examined the between-day repeatability of H-reflex and V-wave measurements during dynamic balance perturbations. The dynamic balance data, measured as COP swaying velocity and COP amplitude, as well as M_max_ values in both standing rest and in all balance perturbation conditions showed no between-day differences, indicating that neural responses were measured in constant conditions. Further, the H-reflex and V-wave methods produced repeatable data during the dynamic balance test. In the future, these methods can be used to investigate adaptation (e.g. training, fatigue, aging) related changes to dynamic balance control.

## Data Availability

The original data is not available as this option was prohibited in the data management plan. As per this plan, the data will also be destroyed by the research team 5 years after the end of the project.
